# *Dicranum motuoense* (Bryophyta): A New Taxon from China, with Special References to Its Complete Organelle Genomes

**DOI:** 10.3390/plants14050650

**Published:** 2025-02-20

**Authors:** Wen-Zhuan Huang, Xin-Yin Ma, Dolgor Y. Tubanova, Oyuna D. Dugarova, Fen-Yao Zhang, Jun Hu, Rui-Liang Zhu, Yu-Huan Wu

**Affiliations:** 1 College of Life and Environmental Sciences, Hangzhou Normal University, Hangzhou 311121, China; wzhmoss@163.com (W.-Z.H.); xinyinma@163.com (X.-Y.M.); 2 Institute of General and Experimental Biology Siberian Branch, Russian Academy of Sciences, UlanUde 670047, Russia; tdolgor@mail.ru (D.Y.T.); otumurova@list.ru (O.D.D.); 3 Forest Resources Monitoring Centre of Zhejiang Province, Hangzhou 310020, China; zhangfenyao163@163.com; 4 CAS Key Laboratory of Mountain Ecological Restoration and Bioresource Utilization, Chengdu Institute of Biology, Chinese Academy of Sciences, Chengdu 610041, China; hujun@cib.ac.cn; 5 Ecological Restoration and Biodiversity Conservation Key Laboratory of Sichuan Province, Chengdu Institute of Biology, Chinese Academy of Sciences, Chengdu 610041, China; 6 Bryology Laboratory, School of Life Sciences, East China Normal University, 500 Dongchuan Road, Shanghai 200241, China

**Keywords:** fragile leaves, mosses, phylogenetic analyses, Himalayas, Shennongjia National Nature Reserve, Yarlung Zangbo Grand Canyon National Nature Reserve

## Abstract

*Dicranum* is one of the most diverse and widespread genera within the family Dicranaceae, encompassing ca. 110 accepted species worldwide. However, the taxonomy of this genus remains notoriously complex, with the circumscription of several species still unresolved, thereby limiting our understanding of the *Dicranum*’s diversity. During a recent survey of *Dicranum* in China, we found an intriguing species characterized by a unique combination of morphological traits including stiff and fragile leaves, sharply denticulate leaf apices, elongated, rectangular and porose laminal cells throughout, bistratose or partially bistratose laminal cells in the distal part, 1–2 stratose alar cells, and a transverse section of the costa in the lower portion of leaf with two stereid bands and undifferentiated epidermal layers. Morphological and molecular phylogenetic analyses, based on five chloroplast markers and one nuclear marker, support the recognition of this moss as a new species, which we described here as *Dicranum motuoense* sp. nov. Furthermore, we present the complete organellar genomes of this newly identified species. The chloroplast genome of *D. motuoense* is 123.94 kb in length, while the mitochondrial genome is 105.77 kb in length. A total of 127 genes and 66 genes were identified in the chloroplast and mitochondrial genomes, respectively. This study not only advances our understanding of species diversity with *Dicranum* but also contributes to the broader knowledge of its evolution. Additionally, a key for the identification of *Dicranum* species with fragile leaves is included.

## 1. Introduction

*Dicranum* Hedw. is one of the most diverse and taxonomically complex genera within the family Dicranaceae, comprising ca. 110 accepted species and over 1250 subordinate taxa [1]. The delimitation of morphological species within this genus remains challenging due to significant variation in gametophytic characteristics, which are heavily influenced by environmental conditions [2,3,4,5]. Recent advances in molecular phylogenetics have facilitated a clearer delineation of certain *Dicranum* species, including those with fragile leaves [6,7], *D. fuscescens* Sm. complexes [8,9], *D. scoparium* Hedw. complexes [4,10], and *D. acutifolium* (Lindb. & Arnell) C.E.O.Jensen complexes [11]. Despite these advancements, our understanding of species diversity within *Dicranum* remains inadequate. A clear illustration of this insufficiency is the ongoing discovery of new species, such as *D. afoninae* Tubanova [12] and *D. baicalense* Tubanova [13] from Russia, *D. hengduanense* W.Z.Huang & R.L.Zhu [7] and *D. shennongjiaense* W.Z.Huang & R.L.Zhu [14] from China, and *D. annapurnaense* W.Z. Huang, T.X.Zheng & Y.Huan Wu. from Nepal [15]. Further studies on the diversity of *Dicranum* species are therefore essential.

To enhance our understanding of *Dicranum* diversity, we conducted extensive surveys in China, especially in the Himalaya region and central China, in recent years [5,7,14,15]. In Hubei and Xizang (Tibet), we found a notable species that undoubtedly belongs to *Dicranum*, characterized by its consistently fragile leaves. This species shares similarities with *D. annapurnaense*, *D. fragilifolium* Lindb., *D. fulvum* Hook., *D. hakkodense* Cardot, *D. hengduanense*, *D. pacificum* Ignatova & Fedosov, *D. tauricum* Sapjegin, and *D. viride* (Sull. & Lesq.) Lindb. [2,3,6,7,15,16,17,18]. However, it exhibits a unique combination of morphological features, including elongated rectangular basal laminal cells with prominent pores, serrulate leaf apices, bistratose or partially bistratose laminal cells in the upper leaf portion, and a lower leaf costa characterized by two stereid bands and undifferentiated abaxial and adaxial epidermal layers. This distinctive morphological combination prompted further investigation.

The analysis of organelle genomes is essential for understanding phylogenetic relationships, evolutionary history, and taxonomy [7,19,20,21]. Recent advancements in sequencing technologies have led to a significant increase in the publication of organelle genomes. As of September 2023, the NCBI database has released approximately 13,000 plastomes and 673 mitogenomes of plants, yet only 285 species have had both genomes assembled [22]. In comparison to seed plants, organellar genomes in bryophytes remain poorly explored. To date, only 49 bryophytes species have had both chloroplast and mitochondrial genomes assembled, including 25 species of mosses, 21 species of liverworts, and 3 species of hornworts (Supplementary Table S1), despite bryophytes comprising approximately 21,000 known species [1]. Clearly, our understanding of organelle genomes in bryophytes is severely limited. Moreover, data on the organelle genome of Dicranaceae are particularly scarce. Currently, the NCBI database contains only three published chloroplast genomes and one mitochondrial genomes from Dicranaceae (https://www.ncbi.nlm.nih.gov/), significantly hindering our understanding of the organelle genomes characteristics within this family.

The primary objectives of this study include (1) to introduce a new species based on both morphological and phylogenetic evidence, (2) to present the complete organelle genomes of this new species, and (3) to provide an updated key to *Dicranum* species with fragile leaves in the Northern Hemisphere.

## 2. Results

### 2.1. Results of Phylogenetic Analyses

**Figure 1:**
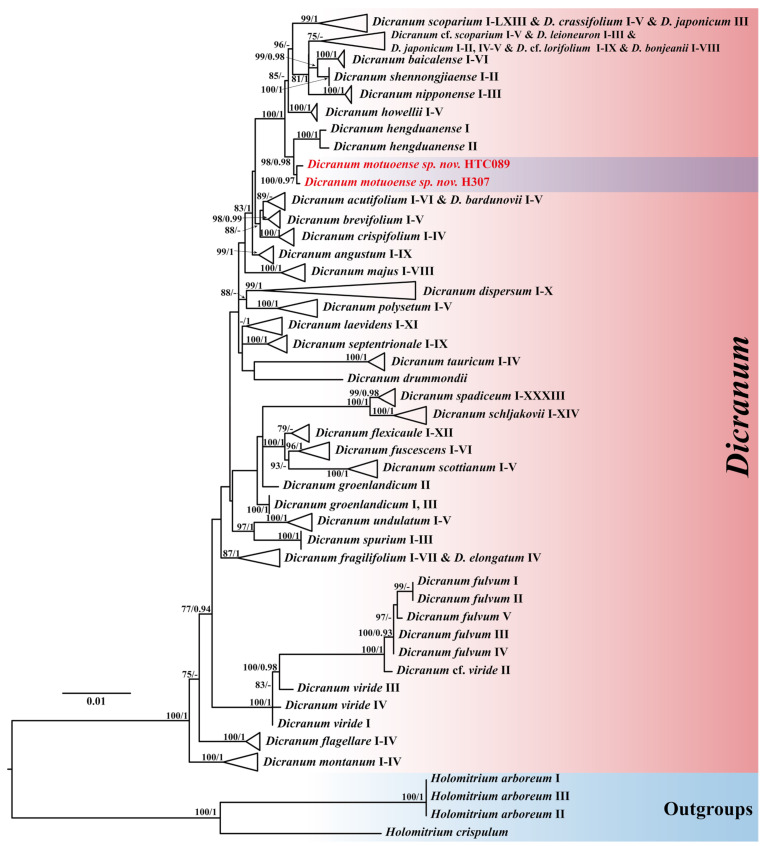
The phylogeny of the *Dicranum* species inferred from the combined dataset (*trn*H-*psb*A, *rps*4-*trn*T, *trn*L-*trn*F, *rps*19-*rpl*2, *rpo*B, and ITS). The topology derived from the best-scoring ML tree in IQtree is shown. ML bootstrap values BS ≥ 70 are shown on the left and Bayesian posterior probabilities values PP ≥ 0.90 on the right. Two newly sequenced accessions are highlighted in red.

The aligned six-loci dataset with 2,731 characters is composed of the following segments and lengths: nrITS1-5.8S-ITS2 (798 bp), *rps*19-*rpl*2 (353 bp), *rpo*B (469 bp), *rps*4-*trn*T (522 bp), *trn*H-*psb*A (139 bp), and *trn*L-*trn*F (450 bp). Among the 2731 aligned nucleotides analyzed across 307 accessions, there were 2167 constant sites, 178 singleton sites, and 386 parsimony-informative sites. Both maximum likelihood (ML) and Bayesian inference (BI) analyses produced congruent trees topologies, with strong support for most nodes. The maximum likelihood tree, annotated with bootstrap values (BS_ML_) and Bayesian posterior probabilities (PP_BI_), is shown in Figure 1.

In the phylogenetic analyses (Figure 1), all *Dicranum* species formed a well-supported clade (BS_ML_ = 100, PP_BI_ = 1). Within the *Dicranum* clade, two newly sequenced *Dicranum* accessions from China formed a distinct clade (BS_ML_ = 100, PP_BI_ = 0.97), which is closely related to the *D. hengduanense* clade (BS_ML_ = 98, PP_BI_ = 0.98).

### 2.2. Taxonomic

**Figure 2:**
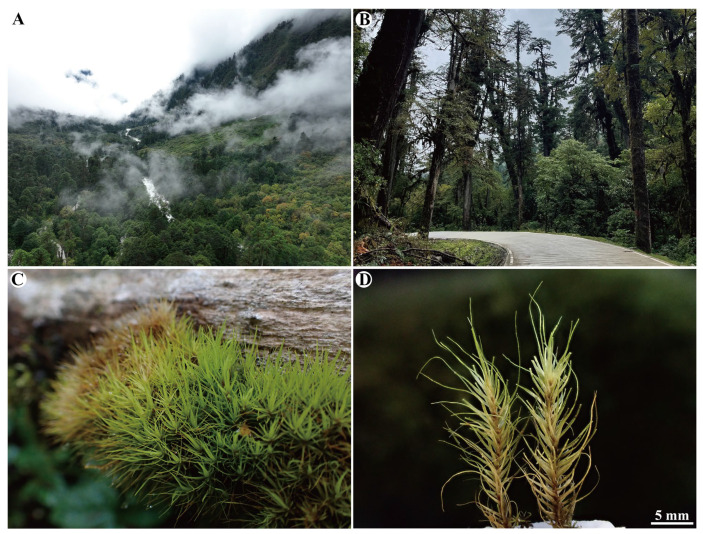
*Dicranum motuoense* W.Z.Huang, Tubanova & Y.Huan Wu. (**A**,**B**) The locality and habitat of the holotype specimen; (**C**) population; (**D**) plants. All from *W.-Z.Huang & F.-Y.Zhang 20241012-77* (holotype: HTC!).

**Figure 3:**
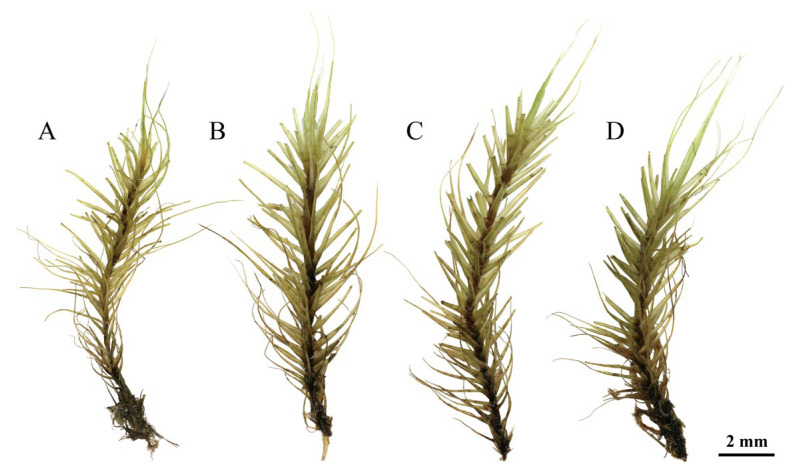
*Dicranum motuoense* W.Z.Huang, Tubanova & Y.Huan Wu. (**A**) Plant when dry; (**B**–**D**) plants when moist. All from *W.-Z.Huang & F.-Y.Zhang 20241012-77* (holotype: HTC!).

**Figure 4:**
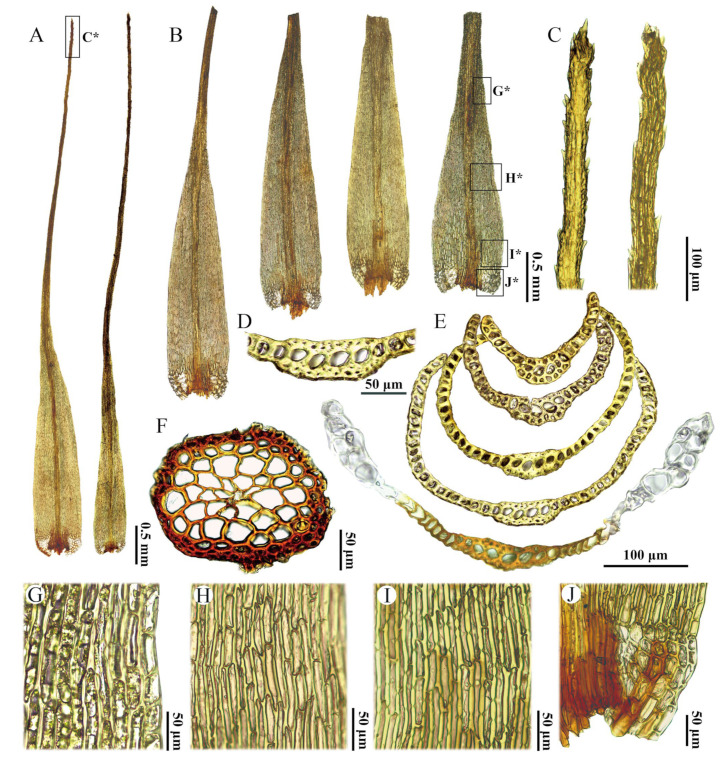
*Dicranum motuoense* W.Z.Huang, Tubanova & Y.Huan Wu. (**A**) Leaves; (**B**) leaves showing tips broken off, (C*,G*,H*,I*,J*) indicates the position of (**C**,**G**,**H**,**I**,**J**) on the leaf, respectively; (**C**) apices; (**D**) the transverse section of the costa in the lower portion of the leaf; (**E**) the transverse sections of the leaf; (**F**) the transverse section of the stem; (**G**) upper laminal cells; (**H**) middle laminal cells; (**I**) basal laminal cells; (**J**) alar cells. All from *W.-Z.Huang & F.-Y.Zhang 20241012-77* (holotype: HTC!).

**Figure 5:**
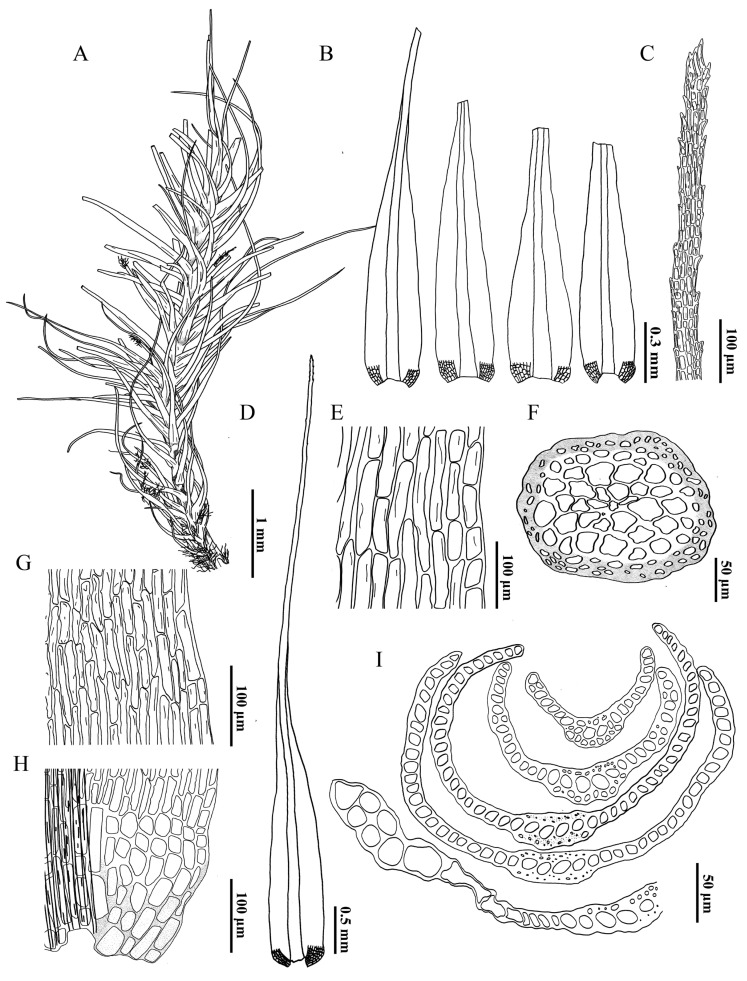
*Dicranum motuoense* W.Z.Huang, Tubanova & Y.Huan Wu. (**A**) Plant; (**B**) leaves showing tips broken off; (**C**) apices; (**D**) leaf; (**E**) upper laminal cells; (**F**) transverse section of stem; (**G**) basal laminal cells; (**H**) alar cells; (**I**) transverse section of stem. All from *W.-Z.Huang & F.-Y.Zhang 20241012-77* (holotype: HTC!).

*Dicranum motuoense W.Z.Huang, Tubanova & Y.Huan Wu, sp. nov.* (Figure 2, Figure 3, Figure 4, Figure 5 and Figures S1 and S2).

Diagnosis. Differing from all other species of the genus Dicranum in the combination of the following characters: invariably fragile leaves; sharply denticulate leaf apices; a costal cross-section in the lower portion with stereid bands on both sides of guide cells, and lacking both abaxial and adaxial epidermal layers; elongated rectangular laminal cells throughout, with prominent pores; and bistratose or portion bistratose laminal cells in the upper leaf portion.

Type: China. Xizang. Linzhi City, Motuo County, Yarlung Zangbo Grand Canyon National Nature Reserve, from Pai Town to Beibeng Village, along Paimo Road, Xiaoyandong, 29°24′56.52″ N, 95°4′7.88″ E, 2691 m, on tree trunks, 12 October 2024, *W.-Z. Huang & F.-Y. Zhang 20241012-77* (holotype: HTC!; isotype: HSNU!, KUN!).

Description. Plants in dense tufts, light green or yellowish green, slightly glossy. Stems 1–2.5 cm long, simple, without or with sparsely white tomentum in upper part, with brownish tomentum near base. Transverse section of stem rounded to oval, epidermis with 1–2 layers of smaller, brown, thick-walled cells, inner cortical cells larger, thin-walled, colorless, central strand cells well differentiated. Leaves straight, erect—spreading when moist, little changed when dry, strongly fragile, with most apices broken off, more rarely apices intact in upper leaves. Leaves lanceolate at base, gradually tapering into a long canaliculated apex, 7.5–9.0 × 0.45–0.65 mm, apex 1.3–2 times as long as the leaf base. Leaf margins sharply denticulate near apex, entire below. Costa strong, occupying ca. 1/7–1/5 of leaf width at base, 90–130 µm wide at leaf base, filling leaf acumen, smooth or slightly rough on dorsal side in distal part, in transverse section in lower leaf with one row of guide cells, two stereid bands, adaxial and abaxial epidermal layers not differentiated, transverse section in the upper portion with one row of guide cells, usually with 1 layer of abaxial epidermal cells and 1 layer of adaxial stereid or substereid cells, sometimes cells with large lumen. Leaf laminal cells smooth, unistratose below, bistratose or bistratose in patches above. Upper laminal cells are elongate-rectangular, 40–90 × 8–19 µm, thick-walled, porose, marginal cells usually short rectangular to subquadrate, 35–60 × 15–20 µm; middle and basal laminal cells elongated and rectangular to linear, 50–130 × 8–12 µm, thick-walled, porose; alar cells differentiated, 1–2 stratose, subquadrate, with slightly thickened walls, brownish or white, not extending to costa, there are few fragile, thin-walled, colorless cells between costa and alar cells. Sporophytes not seen.

Etymology. The epithet “*motuoense*” is named after Motuo County, where the holotype of the new species was found.

Distribution and habitat. *Dicranum motuoense* is currently documented from two distinct localities in China: Motuo County in Tibet (Southwestern China) and the Shennongjia Forestry District in Hubei Province (Central China). This species exhibits a highly specialized habitat preference, growing exclusively on tree trunks (Figure 2B,C) at elevations of 2691 m and 2750 m. Notably, *D. motuoense* is frequently found in association with several bryophytes species, including *Herbertus aduncus* (Dicks.) Gray, *Pelekium versicolor* (Hornsch. ex Müll.Hal.) Touw., *Plagiochila devexa* Steph., and *Tortella fragilis* (Hook. & Wilson) Limpr. These ecological associations suggest a potential niche preference or symbiotic relationship within its microhabitat, which warrants further investigation.

Additional specimen examined. China. Hubei Province. Shennongjia Forestry District, Muyu Town, Shennongjia National Nature Reserve, Banbiyan, 31°27′ N, 110°13′ E, ca. 2750 m, on tree trunk, 25 July 2020, *S.-X.Liu 10504-4* (paratypes: HTC!, CCNU!).

Note. *Dicranum motuoense* is well characterized by the following traits: (1) stiff leaves with apices frequently broken off (Figure 2D, Figure 3, Figure 4B, Figure 5A,B and Figures S1 and S2A); (2) a sharply denticulate leaf apex (Figure 3C, Figure 5C and Figure S2B); (3) a transverse cross-section of the costa in the lower portion featuring two stereid bands on both sides of the guide cells, but not extending above the mid-leaf (Figure 4E, Figure 5I and Figure S2F); (4) undifferentiated adaxial and abaxial epidermal layers in the cross-section of the costa in the lower leaf portion (Figure 4D,E, Figure 5I and Figure S2D,F); (5) unistratose laminal cells below, with bistratose or bistratose patches in the distal part (Figure 4E, Figure 5I and Figure S2F); (6) elongated rectangular laminal cells throughout that are consistently porose (Figure 4G–I, Figure 5E,G,H, and Figure S2G–I); (7) 1–2 stratose alar cells (Figure 4E and Figure S2F), and (8) tree trunks as their ecological habitat (Figure 2C).

### 2.3. Feature of Chloroplast Genomes

**Figure 6:**
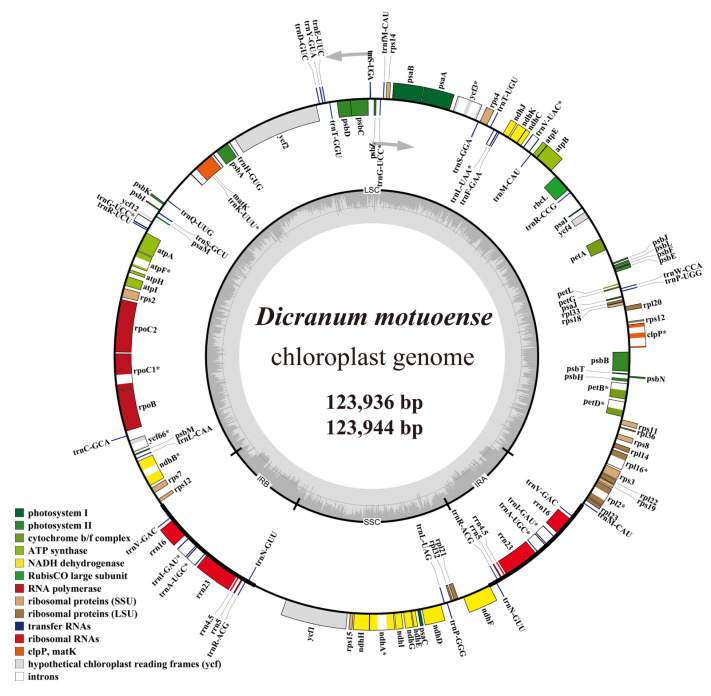
Circular chloroplast genome maps of *Dicranum motuoense*. The asterisk indicates intron-containing genes.

The chloroplast genomes of two *Dicranum motuoense* specimens were 123,936 bp and 123,944 bp in length, respectively. Both genomes exhibited a circular DNA molecule with a typical quadripartite structure, consisting of a large single-copy (LSC) region of 86,174 and 86,182 bp, a small single-copy (SSC) region of 18,582 bp, and a pair of inverted repeats (IRs) of 9500 bp each (Figure 6). The nucleotide composition of both plastomes was highly similar, with 35.0% adenine (A), 35.0% thymine (T), 14.9% guanine (G), and 15.1% cytosine (C), resulting in an overall GC content of 30.0%.

A total of 127 functional genes were annotated in the genomes, including 82 protein-coding genes, eight ribosomal RNAs (rRNAs), and 37 transfer RNA (tRNAs). Among these, nine genes (four rRNAs and five tRNAs) were duplicated in the IR regions (Figure 6; Supplementary Table S2). Additionally, 19 genes were identified as intron-containing, with 17 genes containing a single intron and the *clp*P and *ycf*3 genes each harboring two introns (Figure 6; Supplementary Table S2).

### 2.4. Feature of Mitochondrial Genomes

**Figure 7:**
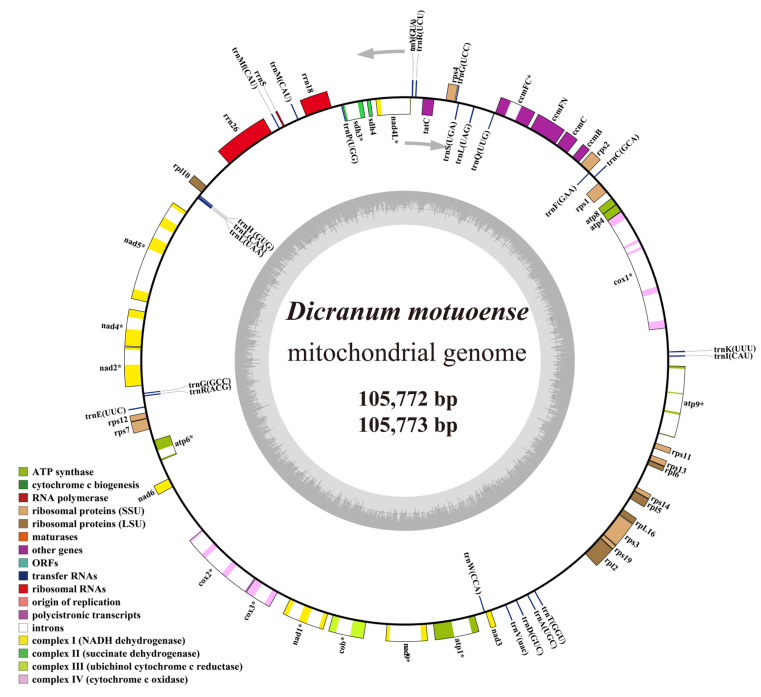
Circular mitochondrial genome maps of *Dicranum motuoense*. The asterisk indicates intron-containing genes.

The mitochondrial genomes of two *Dicranum motuoense* specimens were assembled into a single circular chromosome, measuring 105,772 bp and 105,773 bp in length, respectively, both exhibiting a GC content of 40.5% (Figure 7). These mitogenomes consist of coding regions with a combined length of 31,257 bp, introns measuring 31,301 bp and 31,302 bp, and intergenic spacers totaling 43,214 bp. A total of 66 functional genes were annotated, including three rRNAs, 24 tRNAs, and 39 protein-coding genes (Figure 7; Supplementary Table S3). Among these genes, 15 were identified as intron-containing, with a total of 25 introns distributed across them. Notably, the *cox1* gene harbored the highest number of introns (four), while the *atp*9, *cox*2, and *nad*5 genes each contained three introns (Figure 7; Supplementary Table S3).

### 2.5. Repetitive Sequences and Codon Usage of the Organelle Genomes

**Figure 8:**
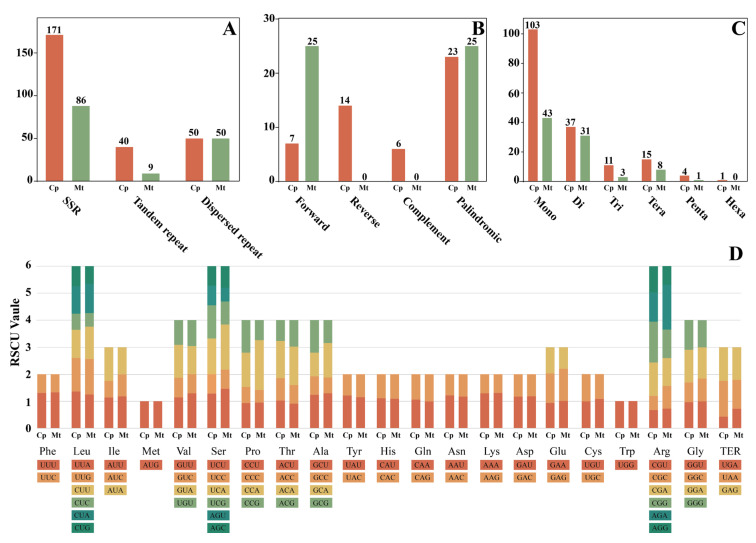
Repetitive sequences and codon usage of chloroplast and mitochondrial genome in *Dicranum motuoense*. (**A**) Repeat types in the organelle genomes; (**B**) dispersed repeat types in the organelle genomes; (**C**) SSR types in the organelle genomes; (**D**) codon usage bias in the organelle genomes. Cp: chloroplast genome; Mt: mitochondrial genome.

A comprehensive analysis of repetitive elements in *Dicranum motuoense* revealed distinct patterns in its chloroplast and mitochondrial genomes. In the chloroplast genome, 171 simple sequence repeats (SSRs), 40 tandem repeats, and 50 dispersed repeats were identified (Figure 8A). Additionally, the chloroplast genome contained 7 forward repeats, 14 reverse repeats, 6 complement repeats, and 23 palindromic repeats. In contrast, the motochondrial genome harbored 86 SSRs, 9 tandem repeats, and 50 dispersed repeats, along with 25 forward repeats and 25 palindromic repeats (Figure 8B).

Among the 171 SSR loci detected in the chloroplast genome, mononucleotide repeats were the most abundant, accounting for 60.23% of all SSRs, followed by dinucleotide (21.64%), tetranucleotide (8.77%), trinucleotide (6.43%), and pentanucleotide repeats (2.34%), with hexanucleotide repeats being the least frequent, represented by only a single site. Similarly, in the mitochindrial genome, 86 SSRs were identified, with mononucleotide repeats constituting 50% of the total, followed by dinucleotide (36.05%), tetranucleotide (9.3%), trinucleotide (3.49%), and pentanucleotide repeats (1.16%) (Figure 8C).

Comparative analysis of the codon usage bias in the coding sequences of 82 chloroplast and 39 mitochondrial identified two codons with a relative synonymous codon usage (RSCU) value of 1: AUG and UGG, encoding methionine (Met) and tryptophan (Trp), respectively (Figure 8D; Supplementary Table S4). In the chloroplast genome, 33 codons exhibited an RSCU > 1, while 29 codons showed the same in the mitochondrial genome. Among these, 11 codons ended with U and 14 with A, indicating a preference for codons terminating with U or A in both organelle genomes of *D. motuoense*. Furthermore, leucine (Leu) was the most frequently encoded amino acid in both genomes, while alanine (Ala) and aspartate (Asp) were the least abundant in the chloroplast and mitochondrial genomes, respectively.

## 3. Discussion

### 3.1. Differentiation with Similar Species

Molecular phylogenetics is a powerful tool for reevaluating traditional taxonomic hypotheses, with monophyly being the main criterion for the phylogenetic species concept [23]. The tree topology derived from our phylogenetic analysis supports the classification of two accessions of *Dicranum motuoense* within the *D. hengduanense* (Figure 1). However, this new species can be readily distinguished from *D. hengduanense* by the following morphological characteristics: (1) leaf margins that are sharply denticulate near the apex (Figure 4C, Figure 5C and Figure S2B), whereas those of *D. hengduanense* are entire [7]; (2) the transverse section of the costa in the lower portion exhibits two stereid bands on both sides of the guide cells in *D. motuoense* (Figure 4D,E, Figure 5I and Figure S2D,F), whereas *D. hengduanense* lacks stereid bands [7]; and (3) upper and median laminal cells characterized by prominent pores (Figure 4G,H, Figure 5E,G and Figure S2G,H), whereas those of *D. hengduanense* are smooth or only very slightly pitted [7].

Additionally, *Dicranum motuoense* may be confused with *D. fulvum* due to shared characteristics such as fragile leaves and predominantly bistratose laminal cells in the upper leaf portion (Figure 4B,E, Figure 5A,B,I and Figure S2A,F) [2,3,18,24,25]. However, our phylogenetic results indicate that these species do not cluster together (Figure 1). Morphologically, *D. motuoense* can be distinguished from *D. fulvum* by the following features: the leaves of *D. motuoense* are moderately fragile and straight when dry (Figure 3 and Figure 5A), whereas those of *D. fulvum* are only occasionally fragile and tend to be crisped when dry [3,6]. Furthermore, the basal laminal cells of *D. motuoense* are elongated and rectangular, measuring 50–120 µm in length, and characterized by prominent pores (Figure 4G,H, Figure 5G and Figure S2G,H), whereas those of *D. fulvum* are short and rectangular, measuring only 20–35 µm in length, and are entirely smooth or possess only a few pits [3,6]. Additionally, the transverse section of the costa in the lower portion of *D. motuoense* lacks differentiated epidermal layers (Figure 4D,E, Figure 5I and Figure S2D,F), whereas the abaxial and adaxial epidermal layers of *D. fulvum* are well differentiated or sometimes only the abaxial epidermis is differentiated [3,6]. Finally, the two species also can be distinguished by the characteristic width of the costa, which occupies ca. 1/7–1/5 of the leaf base in *D. motuoense* (Figure 2A,B and Figure S2A), compared to 1/4–1/3 in *D. fulvum* [3,24].

*Dicranum annapurnaense*, an interesting species recently discovered in the Himalayan region, shares several morphological characteristics with *D. motuoense*, including fragile leaves and elongated and rectangular, porose laminal cells throughout [15]. However, *D. motuoense* can be clearly distinguished by its sharply denticulate leaf apex (Figure 3C, Figure 5C and Figure S2B); a costa in the lower leaf portion that features two stereid bands on both sides of the guide cells (Figure 4E, Figure 5I and Figure S2F); laminal cells that are bistratose or bistratose in patches above the mid-leaf (Figure 4E, Figure 5I and Figure S2F); alar cells with slightly thickened walls, not extending to the costa, and the presence of a few fragile, thin-walled, colorless cells between the costa and alar cells (Figure 4A,B,D, Figure 5B,D,H and Figure S2A,J); and a robust costa occupying ca. 1/7–1/5 of the leaf width at the base. In contrast, *D. annapurnaense* is characterized by leaves that are sometimes caduceus, with residual alar cells on the stem forming a leafless section; a smooth leaf apex; a costa in the lower leaf portion that lacks stereid bands; unistratose laminal cells above the mid-leaf; a slim costa occupying ca. 1/12–1/10 of the leaf width at the base; alar cells with very thick walls; and the absence of fragile, thin-walled, colorless cells between the costa and alar cells [15].

Furthermore, five other species—*D. fragilifolium*, *D. hakkodense*, *D. pacificum*, *D. tauricum*, and *D. viride*—exhibit fragile leaves [2,3,5,6,7,15,18,24] and share similarities with *D. motuoense*. However, these species can be distinguished by the following characteristics: (1) both *D. pacificum* and *D. tauricum* lack stereid bands in the transverse section of the costa in the lower portion of the leaf, have leaf lamina cells with thin or slightly thick walls without pores, and possess unistratose alar cells [2,5,6,7,24]; in contrast, *D. motuoense* possesses two stereid bands on both sides of the guide cells in the lower portion of the leaf (Figure 4E, Figure 5I and Figure S2F), leaf lamina cells with thick, porose walls (Figure 4G–I, Figure 5E,G,H and Figure S2G–I), and 1–2 stratose alar cells (Figure 4E, Figure 5I and Figure S2F). (2) *D. fragilifolium* have nearly entirely or slightly blunt-toothed margins near the apex, and well-differentiated abaxial epidermal cells in the transverse section of the costa in the lower portion of the leaf [2,3,5,6,7,24], whereas the margins near the apex of *D. motuoense* are sharply denticulate (Figure 4C, Figure 5C and Figure S2B), and its abaxial epidermal cells are not differentiated (Figure 4D,E, Figure 5I and Figure S2D,F). (3) *D. hakkodense* is distinguished by its non-porose or rarely porose basal laminal cells, quadrate to short rectangular upper and median lamina cells (up to 35 µm), and well-differentiated adaxial and abaxial epidermal layers in the transverse section of the costa in the lower leaf portion [6]; in contrast, *D. motuoense* has prominently porose basal laminal cells (Figure 4I, Figure 5G and Figure S2I), rectangular to elongated rectangular upper and median lamina cells (up to 90 µm) (Figure 4G,H, Figure 5E,G and Figure S2G,H), and lacks differentiated epidermal cells in the lower part of the leaf (Figure 4D,E, Figure 5I and Figure S2D,F). (4) *D. viride*, which sometimes has leaves with entire margins at the apex, can be distinguished by its nearly quadrate to short rectangular laminal cells and well-differentiated epidermal layers of the costa in the lower portion of the leaf on both sides, or sometimes with only the abaxial epidermis being differentiated [5,6]. In contrast, in *D. motuoense*, the laminal cells are rectangular to elongated rectangular (Figure 4G–I, Figure 5E–G and Figure S2G–I), and the transverse section of the costa in the lower portion lacks differentiated epidermal layers (Figure 4D,E, Figure 5I and Figure S2D,F).

### 3.2. Organelle Genomes

In this study, two newly sequenced plastomes of *Dicranum motuoense* were assembled, measuring 123,936 bp and 123,944 bp in length, respectively, and contained 82 protein-coding genes (Figure 6). These plastomes exhibit sequence lengths and compositions comparable to previously reported plastomes of Dicranaceae species, such as *Chorisodontium aciphyllum* (Hook. f. & Wilson) Broth. [26] and *D. hengduanense* [7]. Moss plastomes display substantial size variability, ranging from 122,213 bp in *Funaria hygrometrica* Hedw. to 149,016 bp in *Takakia lepidozioides* S.Hatt. & Inoue [27]. Previous studies have identified the absence of essential genes as a key factor contributing to the plastome size reduction [28,29]. Consistent with this, our results reveal that *D. motuoense* has lost 10 protein-coding genes compared to *T. lepidozioides*, including *cys*T, *ccs*A, *cys*A, *rps*16, *ycf*10, *rpo*A, *tuf*A, *pet*N, *pbf*1, and *psb*30 [30]. Interestingly, these genes are also absent in most Bryopsida moss plastomes [27], suggesting a common trend of gene loss during moss diversification [28,31].

One of the most prominent features of moss mitogenomes is their highly conserved structural evolution and a general trend of size reduction across the moss phylogenetic tree [32,33,34,35,36,37]. Additionally, previous studies have demonstrated that mitochondrial gene content is the most conserved component across Bryophyta, despite independent gene losses in some lineages [32,37]. For instance, the *nad*7 gene has been independently pseudogenized in four moss lineages: *Tetraphis* Hedw., *Buxbaumia* Hedw., *Pohlia* Hedw., and *Mielichhoferia* Hornsch. [37]. Intriguingly, our findings indicate that *nad*7 is also pseudogenized in *Dicranum motuoense*, like due to inactivation by multiple stop codons within the coding region (Figure 7), representing the fifth moss lineage in which *nad*7 has become pseudogenized. Furthermore, only one mitochondrial genome from the Dicranales order has been published to data [38]. The mitogenome of *D. motuoense* presented in this study is the second complete mitogenome of Dicranales and the first complete mitogenome of the genus *Dicranum*.

Repetitive sequences are ubiquitous in organelle genomes, playing crucial roles in protecting coding sequences [39] and maintaining genome stability. Simple sequence repeats (SSRs) in organelle genomes are highly polymorphic and are widely utilized as molecular markers in variety identification and other studies [39,40,41,42,43]. This study investigated dispersed and tandem repeats, revealing that tandem repeats are more prevalent in the plastid genome, while dispersed repeats are consistent across organelle genomes (Figure 8A). Additionally, mononucleotide repeats were the most abundant motifs, followed by dinucleotide repeats (Figure 8C), consistent with previous studies on SSRs in other bryophyte organelle genomes [27,32,44]. These SSRs provide valuable candidate molecular markers for *Dicranum*, which can be utiluzed in population genetics, evolutionary studies, molecular breeding, and conservation efforts.

### 3.3. Key to Species of Dicranum with Fragile Leaves in the Northern Hemisphere

1. Leaf apices entire or with few blunt teeth.....................................................................21. Leaf apices sharply denticulate or serrulate..................................................................62. Basal laminal cells nearly quadrate to short rectangular, 20–35(–45) μm long .......................................................................... *D. viride* (Sull. & Lesq.) Lindb. (in part)2. Basal laminal cells elongated and rectangular, 30–100(–120) μm long........................33. Costa in the lower portion of the leaf with stereid bands, sometimes weak, with 2–3 (–4) layers of cells above and below guide cells........................... *D. fragilifolium* Lindb.3. Costa in the lower portion of the leaf lacking stereid bands, with (0–)1–2 layers of cells above and below guide cells.....................................................................................................44. Alar cells unistratose............................................................. *D. tauricum* Sapjegin (in part)4. Alar cells bistratose or 1–2 stratose.......................................................................................55. Upper and middle laminal cells strongly porose; alar cells with thick walls..................... .........................................*D. annapurnaense* W.Z.Huang, T.X.Zheng & Y.Huan Wu5. Upper and middle laminal cells not or very slightly pitted; alar cells with thin walls....... .............................................................*D. hengduanense* W.Z.Huang & R.L.Zhu6. Costa in the lower portion of the leaf with stereid bands; leaf lamina cells with thick walls, sometimes with bulging.................................................................................76. Costa in the lower portion of the leaf without stereid bands, with substereids on both sides of guide cells; leaf lamina cells with thin or slightly thick walls.....................107. The epidermal layers of the costa in the lower portion of the leaf undifferentiated; upper laminal cells elongated and rectangular, 40–90 μm long, with prominent pores .......................*.................... D. motuoense* W.Z.Huang, Tubanova & Y.Huan Wu7. The epidermal layers of the costa in the lower portion of the leaf, well differentiated on both sides, or sometimes with only the abaxial epidermis differentiated; upper laminal cells regularly quadrate to short rectangular, up to 35 μm long, not pitted.............88. Costa abruptly differentiated, semicircular in the transverse section, especially in the middle and proximal parts; cell walls between lamina cells without bulging or slightly bulging.................................................................. *D. viride* (Sull. & Lesq.) Lindb. (in part)8. Costa gradually differentiated, flattened in the transverse section along the entire length; cell walls between lamina cells slightly or strongly bulging................................99. Leaves weakly fragile, some leaf tips broken off; costa broader, occupying 1/3 or more of the total leaf base width.............................................................................*D. fulvum* Hook.9. Leaves fragile; costa narrower, occupying less than 1/3 the leaf base width ............... ................................................................................................................................................. ......................................................................*D. hakkodense* Cardot10. Basal laminal cells linear, 40–80(–120) μm long, with non-porose or slightly porose walls........................................................................................ *D. tauricum* Sapjegin (in part)10. Basal laminal cells rectangular, 20–40(–50) μm long, with smooth walls................ .............................................................................*D. pacificum Ignatova & Fedoso*

## 4. Materials and Methods

### 4.1. Taxon Sampling

The infrageneric relationships within the genus *Dicranum* have been well resolved through combined analyses of five plastid loci (*rps*4–*trn*T, *trn*L–*trn*F, *trn*H–*psb*A, *rps*19–*rpl*2, and *rpo*B) along with the nrITS1–5.8S–ITS2 region [4,5,7,11,15,45,46]. In this study, two samples were collected from China. The holotype specimen (*W.-Z.Huang & F.-Y.Zhang 20241012-77*) was collected from an alpine coniferous forest in Motuo County, Tibet, dominated by *Abies delavayi* Diels, *Abies delavayi* var. *motuoensis* Cheng et L.K.Fu, and *Tsuga dumosa* (D.Don) Eichler (Figure 2A,B). Upon examining our *Dicranum* collection in China, a paratype specimen (*S.-X.Liu 10504-4*) was identified from the Shengnongjia National Nature Reserve, Hubei Province, China. Both samples were included in the phylogenetic analysis to determine the placement of this moss. Three accessions of *Holomitrium arboretum* Mitt. and one accession of *H. crispulum* Mart. were selected as outgroups. Additionally, two specimens of *D. fulvum* from the U.S.A. and three from Russia were also sequenced. The remaining 296 *Dicranum* accessions were obtained from GenBank. A detailed list of taxa, including collection localities, vouchers, herbarium codes, and GenBank accession numbers, is provided in Supplementary Table S5.

### 4.2. Morphological Study

The plant photos were taken using a digital camera (Olympus TG6; Olympus, Tokyo, Japan). A Leica stereo zoom scope (Leica EZ4; Leica, Wetzlar, Germany) and an Olympus microscope (Olympus BX51; Olympus, Tokyo, Japan) were used to examine the specimens, and microscopic images were captured by a digital camera (MOTICAM S6; Motic, Xiamen, China) attached to the microscope. The plant pictures were taken using a stereo microscope (Keyence VHX-6000; Keyence, Osaka, Japan).

### 4.3. DNA Extraction, Sequencing, Assembly and Annotation

Sample preparation and DNA extraction followed protocols used in previous studies [20,32]. High-quality genomic DNA from each sample was used for the whole genome sequencing to obtain paired-end 150 bp raw reads on the Novaseq-SE50 platform (Novogene, Tianjin, China) according to the manufacturer’s procedures. Raw reads with a Phred score lower than 30 were removed, retaining high-quality sequences for nuclear DNA and complete circular organelle genome assembly using the GetOrganelle v 1.7.7.1 [47]. Genomes were automatically annotated with CPGAVAS2 [48] and subsequently refined using Geneious v.11.0.3 [49], with *Dicranim hengduanense* (accession number: NC_080897) as the reference plastome and *Chorisodontium aciphyllum* (accession number: MK651511) as the reference mitochondrial genome. Circular organelle genomes maps were drawn using OrganellarGenome DRAW [50]. The newly assembled chloroplast (accession numbers: PQ821713 and PQ821714) and mitochondrial (accession numbers: PQ821739 and PQ821740) genomes were deposited in GenBank. The assembled nuclear data were aligned with published data using *Dicranum scoparium* as a reference (nrITS1-5.8S-ITS2 accession number, KF423564) in Geneious version 11.1.5 [49] and then annotated and extracted.

### 4.4. Repetitive Sequences, and Codon Usage Preference Analyses

Dispersed repeat sequences in the organelle genomes of the holotype specimen (*W.-Z.Huang & F.-Y.Zhang 20241012-77*) were predicted using REPuter [51]. Forward, reverse, palindromic, and complement repeat sequences were identified with the following parameters: length of repeat unit ≥30 bp, sequence consistency ≥90% (Hamming distance = 3). Tandem repeat sequences were predicted using the Tandom Repeats Finder (TRF) web server (https://tandem.bu.edu/trf/trf.html (accessed on 25 December 2024)) [52]. Simple sequence repeats (SSRs) were identified using MISA [53], with minimum repetition threshold values for mono-, di-, tri-, tetra-, penta-, and hexa-nucleotide were set to 10, 5, 4, 3, 3, and 3, respectively. Codon usage bias and relative synonymous codon usage (RSCU) frequencies were calculated using CodonW software (http://codonw.sourceforge.net/ (accessed on 25 December 2024)).

### 4.5. Phylogenetic Analyses

Six sequences were aligned using MAFFT v7.311 [54] and ambiguous alignment regions were trimmed using trimAl v1.2 [55] and manually adjusted. The resulting individual alignments were concatenated in Geneious ver. 11.1.5 [49], with absent data coded as missing.

Phylogenetic analyses were conducted using the maximum likelihood (ML) and Bayesian inference (BI) methods in IQtree version 2.0.6 [56] and MrBayes 3.2.6 [57], respectively. IQtree was performed with the best-fitting substitution model for each DNA region (HKY+F+G4 for ITS-partition, HKY+F+I+G4 for rps19-rpl2-partition, rpoB-partition, rps4-trnT-partition, trnL-trnF-partition, and trnH-psbA-partition) selected by ModelFinder according to the Bayesian information criterion (BIC) [58,59], and the fast bootstrap option with 1000 replicates. For BI analyses, each DNA region was also assigned its own substitution model (HKY+I+G is the for best-fit model for ITS-partition, *rpo*B-partition and *rps*19-*rpl*2-partition; HKY+G for *rps*4-*trn*T-partition and *trn*H-*psb*A-partition; and GTR+I+G for trnL-trnF-partition), as determined by the Akaike information criterion (AIC) [58,59]. Two independent analyses consisting of four Markov chain Monte Carlo (MCMC) chains were run for 5,000,000 generations, with one tree sampled for every 1000 generations. The posterior distribution of the trees was summarized by a >50% majority-rule consensus tree after discarding the first 25% of samples as burn-in. Convergence was assessed by examining the likelihood plots in Tracer v.1.7 [60].

## 5. Conclusions

In this study, a new species, *Dicranum motuoense* sp. nov., is described based on both morphological and molecular evidence from China. This new species is characterized by its stiff and fragile leaves, sharply denticulate apices, elongated rectangular and porose laminal cells throughout, bistratose or bistratose patch laminal cells in the distal part, bistratose alar cells, and the transverse section of the costa in the lower portion of the leaf featuring two stereid bands and undifferentiated epidermal layers. Given the rich diversity of Motuo County and the lack of a comprehensive survey of bryophytes, it is likely that further research on bryophytes in this region will reveal more new species. Additionally, this study introduced the organelle genomes of *D. motuoense*, representing the second chloroplast genome and the first mitochondrial genome of this genus, providing a reference for further research into relationships and boundaries among genera within the Dicranaceae family. Furthermore, genome characteristics were analyzed, revealing that the organelle genomes of *D. motuoense* display an astoundingly conserved structure with other moss species, as reflected in genome size, gene content, repetitive sequences, and codon usage preference. Finally, a key for the identification species of *Dicranum* with fragile leaves is provided.

## Data Availability

Data are contained within the article and Supplementary Materials.

## Supplementary Materials

The following supporting information can be downloaded at: https://www.mdpi.com/article/10.3390/plants14050650/s1, Figure S1: Plants of *Dicranum motuoense* W.Z. Huang, Tubanova & Y.Huan Wu. All from *S.-X.Liu 10504-4* (paratype: HTC!); Figure S2: *Dicranum motuoense* W.Z. Huang, Tubanova & Y.Huan Wu. (**A**) Leaves; (**B**) apices; (**C**) fallen leaf tips with rhizoids at the base; (**D**) the transverse section of the costa in the lower portion of leaf; (**E**) transverse sections of the leaf; (**F**) the transverse section of the stem; (**G**) the upper laminal cells of the leaf; (**H**) middle laminal cells of the leaf; (**I**) basal laminal cells of the leaf; (**J**) alar cells. All from *S.-X.Liu 10504-4* (paratype: HTC!); Table S1: Bryophytes known for assembling both chloroplast and mitochondrial genomes; Table S2: A list of genes in the plastome of *Dicranum motuoense*. *: intron number; Gene (×2): number of copies of multi-copy genes; Table S3: A list of genes in the mitogenome of *Dicranum motuoense*. *: intron number; Gene (×2): number of copies of multi-copy genes; Table S4: Relative synonymous codon usage (RSCU) of the *Dciranum motuoense* organelle genomes. Table S5: Sequences download from GenBank, including taxa, localities, vouchers, herbarium codes, and GenBank accession numbers (*rps*4-*trn*T, *trn*L-*trn*F, *trn*H-*psb*A, *rps*19-*rpl*2, *rpo*B, and ITS). “—” means data missing; newly sequenced specimens are set in boldface.
