# Supplementary material for: Dicranum motuoense (Bryophyta): A New Taxon from China, with Special References to Its Complete Organelle Genomes
Source: Plants (Basel). 2025 Feb 20;14(5):650. doi: 10.3390/plants14050650 (PMC11901946; doi:10.3390/plants14050650)
Supplement: Supplementary file 1 [file plants-14-00650-s001.zip › Supplementary Table S1.pdf]

Supplementary Table S1. Bryophytes known for assembled both chloroplast and mitochondrial genomes.

No.	Species	Family	Plastome/Mitogenome accession Number
**Bryophyta**			
1	*Andreaea regularis*	Adreaceae	ON768814/OP067134
2	*Anomodon rugelii*	Anomodontaceae	ON310497/JF973314
3	*Atrichum angustatum*	Polytrichaceae	MW556444/KC784956
4	*Bartramia pomiformis*	Bartramiaceae	MT024676/KC784955
5	*Buxbaumia aphylla*	Buxbaumiaceae	MN496310/KC784954
6	*Callicladium imponens*	Callicladiaceae	MW822169/KC784951
7	*Climacium dendroides*	Climaciaceae	MT006132/MN942036
8	*Funaria hygrometrica*	Funariaceae	MW648546/KC784959
9	*Myurella julacea*	Plagiotheciaceae	MT809490/MT850126
10	*Orthotrichum obtusifolium*	Orthotrichaceae	KP765733/KX702279
11	*Orthotrichum stellatum*	Orthotrichaceae	MW822170/KC784958
12	*Physcomitrium patens*	Funariaceae	KY126308/AB251495
13	*Pohlia nutans*	Mniaceae	MN937553/MN956803
14	*Polytrichum commune*	Polytrichaceae	MW528408/MG214794
15	*Pseudanomodon attenuatus*	Neckeraceae	MW528223/JX402749
16	*Ptychomnion cygnisetum*	Ptychomniaceae	MW822171/KC784949
17	*Rhodobryum laxelimbatum*	Bryaceae	MW147233/OL754429
18	*Sanionia uncinata*	Scorpidiaceae	KM111545/KP984757
19	*Sphagnum palustre*	Sphagnaceae	KU726621/KC784957
20	*Sphagnum subsecundum*	Sphagnaceae	MW528200/MW528196
21	*Stoneobryum bunyaense*	Orthotrichaceae	MK521875.1/ KX578031
22	*Syntrichia filaris*	Pottiaceae	MK852705/KP984758
23	*Tetraphis pellucida*	Tetraphidaceae	KJ817846/KJ817845
24	*Tortula atrovirens*	Pottiaceae	PP190927/PP212810
25	*Ulota hutchinsiae*	Orthotrichaceae	MW822174/KC784952
**Marchantiophyta**			
26	*Aneura maxima*	Aneuraceae	OQ700947/OQ884166
27	*Aneura pinguis*	Aneuraceae	KY702720/KP728938
28	*Apopellia endiviifolia*	Pelliaceae	JX827163/OQ236451
29	*Calypogeia suecica*	Calypogeiaceae	OP526750/MF401631
30	*Cyathodium cavernarum*	Calypogeiaceae	PP078733/PP078732
31	*Diplophyllum taxifolium*	Scapaniaceae	MT948954/MW091501
32	*Douinia plicata*	Scapaniaceae	MT898431/MW091500
33	*Dumortiera hirsuta*	Dumortieraceae	MH355546/MK284524
34	*Fossombronia foveolata*	Fossombroniaceae	OQ280813/MK749462
35	*Gymnomitrion concinnatum*	Gymnomitriaceae	MH705066/MH705065
36	*Leiosporoceros dussii*	Leiosporocerotaceae	MH577299/MH577300
37	*Marchantia paleacea*	Marchantiaceae	X04465/M68929
38	*Marchantia polymorpha* subsp. *ruderalis*	Marchantiaceae	LC192146/LC192147

39	*Moerckia flotoviana*	Moerckiaceae	OQ280814/OQ236461
40	*Nowellia curvifolia*	Cephaloziaceae	MW528215/MW528214
41	*Pellia epiphylla*	Pelliaceae	OQ280821/OQ236463
42	*Pellia neesiana*	Pelliaceae	OQ280829/OQ236470
43	*Riccia fluitans*	Ricciaceae	MK645896/MK749459
44	*Scapania ampliata*	Scapaniaceae	MT644123/MT755612
45	*Scapania undulata*	Scapaniaceae	OK662585/OR220800
46	*Wiesnerella denudata*	Wiesnerellaceae	MT712073/MT745951
**Anthocerotophyta**			
47	*Anthoceros agrestis*	Anthocerotaceae	MN544311/MN544313
48	*Anthoceros angustus*	Anthocerotaceae	AB086179/MG029262
49	*Anthoceros punctatus*	Anthocerotaceae	MN544310/MN544312
