# Supplementary material for: Dicranum motuoense (Bryophyta): A New Taxon from China, with Special References to Its Complete Organelle Genomes
Source: Plants (Basel). 2025 Feb 20;14(5):650. doi: 10.3390/plants14050650 (PMC11901946; doi:10.3390/plants14050650)
Supplement: Supplementary file 1 [file plants-14-00650-s001.zip › Supplementary Table S2.pdf]

Supplementary Table S2. List of genes in the plastome of *Dicranum motuoense*. *: intron number; Gene (×2): Number of copies of multi-copy genes

**Category**	**Group**	**Gene**
Photosynthesis-related genes	Rubisco	*rbc*L
	Photosystem I	*psa*A, *psa*B, *psa*C, *psa*I, *psa*J, *psa*M
	Photosystem II	*psb*A, *psb*B, *psb*C, *psb*D, *psb*E, *psb*F, *psb*H, *psb*I, *psb*J, *psb*K, *psb*L, *psb*M, *psb*N, *psb*T, *psb*Z
	ATP Synthase	*atp*A, *atp*B, *atp*E, *atp*F*, *atp*H, *atp*I
	Cytochrome b/f complex	*pet*A, *pet*B*, *pet*D*, *pet*G, *pet*L
	NADPH dehydrogenase	*ndh*A*, *ndh*B*, *ndh*C, *ndh*D, *ndh*E, *ndh*F, *ndh*G, *ndh*H, *ndh*I, *ndh*J, *ndh*K
	Chlorophyll biosynthesis	*chl*B, *chl*L, *chl*N
Transcription and translation related genes	Transcription	*rpo*B, *rpo*C1*, *rpo*C2
	Ribosomal proteins	*rps*2, *rps*3, *rps*4, *rps*7, *rps*8, *rps*11, *rps*12, *rps*14, *rps*15, *rps*18, *rps*19, *rpl*2*, *rpl*14, *rpl*16*, *rpl*20, *rpl*21, *rpl*22, *rpl*23, *rpl*32, *rpl*33, *rpl*36
	Translation initiation factor	*inf*A
RNA genes	Ribosomal RNA	rrn5(×2), rrn4.5(×2), rrn16(×2), rrn23(×2)
	Transfer RNA	*trn*A-UGC(×2)*, *trn*C-GCA, *trn*D-GUC, *trn*E-UUC, *trn*F-GAA, *trnf*M-CAU, *trn*G-UCC*, trnG-UCC, *trn*H-GUG, *trn*I-GAU (×2)*, *trn*K-UUU*, *trn*L-CAG, *trn*L-CAA, *trn*L-UAA*, *trn*M-CAU (×2), *trn*N-GUU (×2), *trn*P-GGG, *trn*P-UGG, *trn*Q-UUG, *trn*R-ACG (×2), *trn*R-CCG, *trn*R-UCU, *trn*S-GCU, *trn*S-GGA, *trn*S-UGA, *trn*T-GGU, *trn*T-UGU, *trn*V-GAC (×2), *trn*V-UAC*, *trn*W-CCA, *trn*Y-GUA
Other genes	RNA processing	*mat*K
	Carbon metabolism	*cem*A
	Fatty acid synthesis	*acc*D
	Proteolysis	*clp*P**
Genes of unknown function	Conserved reading frame	*ycf*1, *ycf*3**, *ycf*4, *ycf*12, *ycf*66*
