# Supplementary material for: Dicranum motuoense (Bryophyta): A New Taxon from China, with Special References to Its Complete Organelle Genomes
Source: Plants (Basel). 2025 Feb 20;14(5):650. doi: 10.3390/plants14050650 (PMC11901946; doi:10.3390/plants14050650)
Supplement: Supplementary file 1 [file plants-14-00650-s001.zip › Supplementary Table S3.pdf]

Supplementary Table S3. List of genes in the mitogenome of *Dicranum motuoense*.

*: intron number; Gene (×2): Number of copies of multi-copy genes;

Category	Group	Gene
Protein-coding genes	Complex I	*nad*1**, *nad*2*, *nad*3, *nad*4*, *nad*4L*, *nad*5***, *nad*6, *nad*9*
	Complex II	*sdh*3*, *sdh*4
	Complex III	*cob**
	Complex IV	*cox*1****, *cox*2***, *cox*3*
	Complex V	*atp*1*, *atp*4, *atp*6* , *atp*8, *atp*9***
	Cytochrome c biogenesis	*ccm*B, *ccm*C, *ccm*FC*, *ccm*FN
	Large subunit of ribosome	*rpl*2, *rpl*5, *rpl*6, *rpl*10, *rpl*16
	Small subunit of ribosome	*rps*1, *rps*2, *rps*3, *rps*4, *rps*7, *rps*11*, *rps*12, *rps*13, *rps*14, *rps*19
	protein transport	*tat*C
Ribosomal RNAs		*rrn*5, *rrn*18, *rrn*26
Transfer RNAs		*trn*A-UGC, *trn*C-GCA, *trn*D-GUC, *trn*E-UUC, *trn*F-GAA, *trn*G-GCC, *trn*G-UCC, *trn*H-GUG, *trn*I-CAU, *trn*K-UUU, *trn*L-CAA, *trn*L-UAA, *trn*L-UAG, *trn*M-CAU, *trn*Mf-CAU, *trn*P-UGG, *trn*Q-UUG, *trn*R-ACG, *trn*R-UCU, *trn*S-UGA, *trn*T-GGU, *trn*V-UAC, *trn*W-CCA, *trn*Y-GUA
