# Supplementary material for: Dicranum motuoense (Bryophyta): A New Taxon from China, with Special References to Its Complete Organelle Genomes
Source: Plants (Basel). 2025 Feb 20;14(5):650. doi: 10.3390/plants14050650 (PMC11901946; doi:10.3390/plants14050650)
Supplement: Supplementary file 1 [file plants-14-00650-s001.zip › Supplementary Table S4.pdf]

Supplementary Table S4. Relative synonymous codon usage (RSCU) of the *Dciranum motuoense* organelle genomes.

**chloroplast genome**				**mitochondrial genome**			
**Symbol**	**Codon**	**No.**	**RSCU**	**Symbol**	**Codon**	**No.**	**RSCU**
Ala	GCU	88	1.24	Ala	GCU	96	1.29
Ala	GCC	48	0.68	Ala	GCC	44	0.59
Ala	GCA	62	0.88	Ala	GCA	94	1.27
Ala	GCG	85	1.2	Ala	GCG	63	0.85
Arg	CGU	57	0.67	Arg	CGU	48	0.72
Arg	CGC	44	0.52	Arg	CGC	56	0.84
Arg	CGA	105	1.24	Arg	CGA	69	1.03
Arg	CGG	128	1.51	Arg	CGG	71	1.06
Arg	AGA	91	1.07	Arg	AGA	110	1.65
Arg	AGG	83	0.98	Arg	AGG	47	0.7
Asn	AAU	720	1.22	Asn	AAU	229	1.17
Asn	AAC	461	0.78	Asn	AAC	162	0.83
Asp	GAU	258	1.17	Asp	GAU	99	1.18
Asp	GAC	183	0.83	Asp	GAC	69	0.82
Cys	UGU	149	0.99	Cys	UGU	147	1.08
Cys	UGC	153	1.01	Cys	UGC	125	0.92
Gln	CAA	525	1.06	Gln	CAA	189	0.99
Gln	CAG	461	0.94	Gln	CAG	191	1.01
Glu	GAA	420	1.07	Glu	GAA	182	1.19
Glu	GAG	363	0.93	Glu	GAG	123	0.81
Gly	GGU	145	0.96	Gly	GGU	80	0.99
Gly	GGC	110	0.73	Gly	GGC	68	0.84
Gly	GGA	183	1.21	Gly	GGA	94	1.16
Gly	GGG	166	1.1	Gly	GGG	81	1
His	CAU	290	1.11	His	CAU	140	1.09
His	CAC	234	0.89	His	CAC	118	0.91
Ile	AUU	918	1.14	Ile	AUU	302	1.18
Ile	AUC	488	0.61	Ile	AUC	207	0.81
Ile	AUA	1004	1.25	Ile	AUA	260	1.01
Leu	UUA	923	1.36	Leu	UUA	332	1.25
Leu	UUG	844	1.24	Leu	UUG	348	1.31
Leu	CUU	710	1.04	Leu	CUU	321	1.2
Leu	CUC	408	0.6	Leu	CUC	133	0.5
Leu	CUA	684	1.01	Leu	CUA	290	1.09
Leu	CUG	509	0.75	Leu	CUG	175	0.66
Lys	AAA	1535	1.29	Lys	AAA	447	1.31
Lys	AAG	853	0.71	Lys	AAG	238	0.69
Met	AUG	660	1	Met	AUG	272	1
Phe	UUU	1179	1.31	Phe	UUU	608	1.33
Phe	UUC	622	0.69	Phe	UUC	309	0.67

Pro	CCU	100	0.93	Pro	CCU	93	0.95
Pro	CCC	64	0.6	Pro	CCC	46	0.47
Pro	CCA	136	1.27	Pro	CCA	180	1.84
Pro	CCG	129	1.2	Pro	CCG	72	0.74
Ser	UCU	145	1.28	Ser	UCU	134	1.46
Ser	UCC	82	0.72	Ser	UCC	65	0.71
Ser	UCA	149	1.32	Ser	UCA	153	1.67
Ser	UCG	139	1.23	Ser	UCG	78	0.85
Ser	AGU	81	0.72	Ser	AGU	47	0.51
Ser	AGC	83	0.73	Ser	AGC	73	0.8
TER	UAA	294	0.43	TER	UAA	197	0.72
TER	UAG	910	1.32	TER	UAG	293	1.07
TER	UGA	862	1.25	TER	UGA	328	1.2
Thr	ACU	108	1.03	Thr	ACU	59	0.91
Thr	ACC	86	0.82	Thr	ACC	45	0.69
Thr	ACA	144	1.38	Thr	ACA	92	1.42
Thr	ACG	80	0.77	Thr	ACG	64	0.98
Trp	UGG	291	1	Trp	UGG	206	1
Tyr	UAU	655	1.21	Tyr	UAU	266	1.15
Tyr	UAC	429	0.79	Tyr	UAC	197	0.85
Val	GUU	440	1.14	Val	GUU	223	1.29
Val	GUC	282	0.73	Val	GUC	123	0.71
Val	GUA	471	1.22	Val	GUA	180	1.04
Val	GUG	350	0.91	Val	GUG	167	0.96
