# Supplementary figures and images for: Dicranum motuoense (Bryophyta): A New Taxon from China, with Special References to Its Complete Organelle Genomes
Source: Plants (Basel). 2025 Feb 20;14(5):650. doi: 10.3390/plants14050650 (PMC11901946; doi:10.3390/plants14050650)

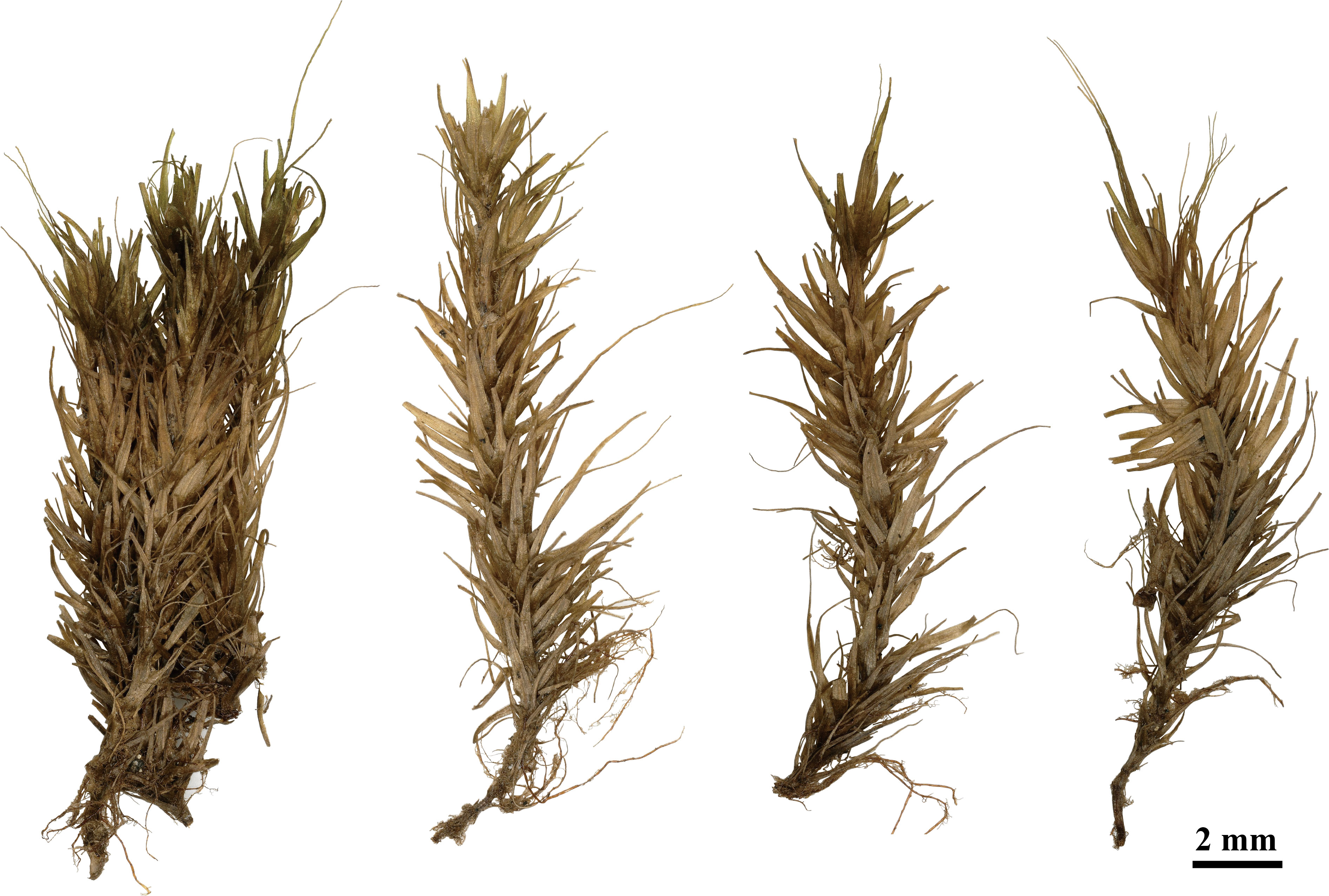

Supplement: Supplementary file 1 [file plants-14-00650-s001.zip › Figure S1.jpg]

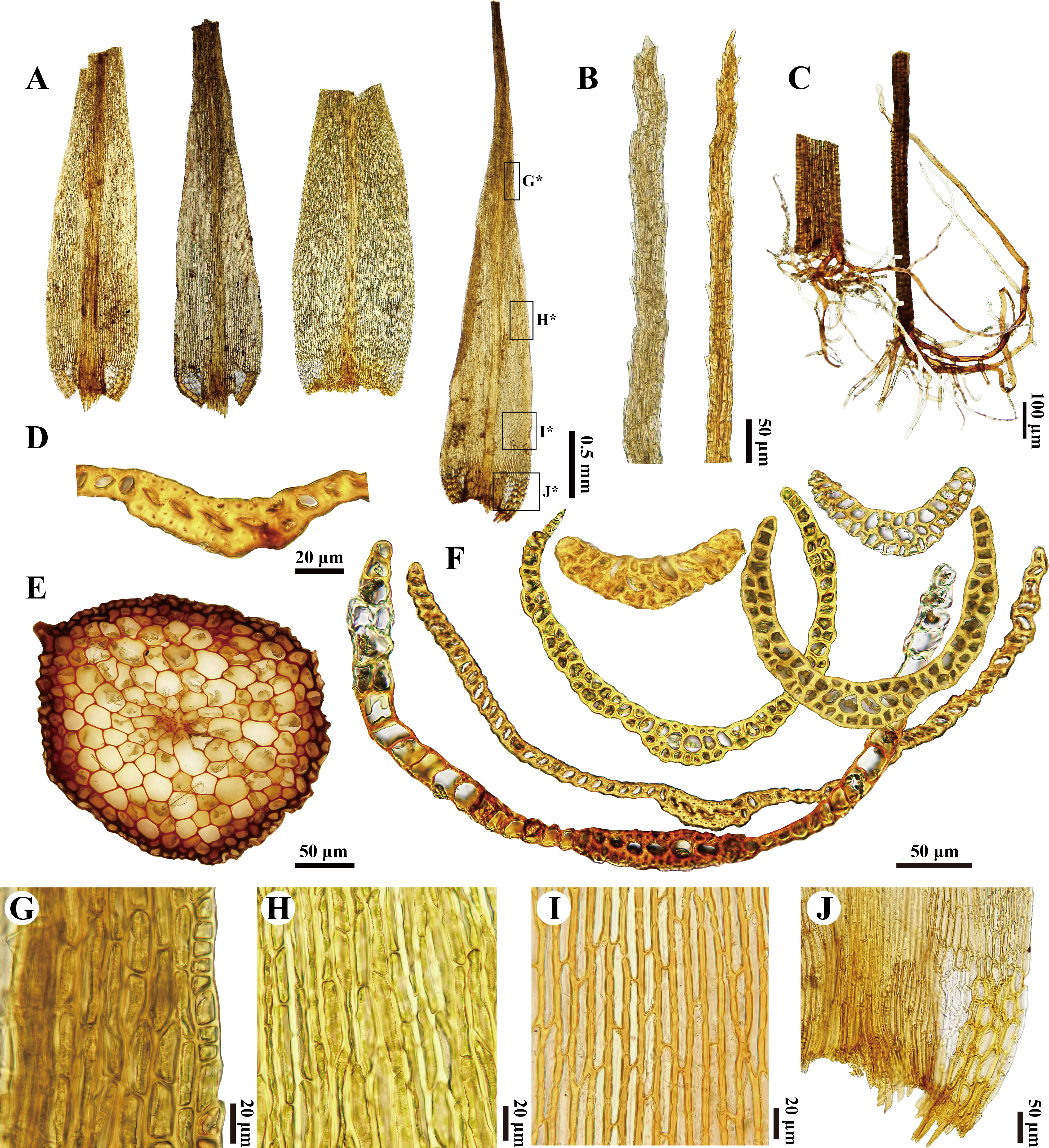

Supplement: Supplementary file 1 [file plants-14-00650-s001.zip › Figure S2.jpg]
